# mRCat: A Novel CatBoost Predictor for the Binary Classification of mRNA Subcellular Localization by Fusing Large Language Model Representation and Sequence Features

**DOI:** 10.3390/biom14070767

**Published:** 2024-06-27

**Authors:** Xiao Wang, Lixiang Yang, Rong Wang

**Affiliations:** 1 School of Computer Science and Technology, Zhengzhou University of Light Industry, Zhengzhou 450002, China; yanglixiang@email.zzuli.edu.cn; 2 Henan Provincial Key Laboratory of Data Intelligence for Food Safety, Zhengzhou University of Light Industry, Zhengzhou 450002, China; 3 School of Electronic Information, Zhengzhou University of Light Industry, Zhengzhou 450002, China; wangrong@zzuli.edu.cn

**Keywords:** mRNAs, subcellular localization, gradient boosting tree, large language model

## Abstract

The subcellular localization of messenger RNAs (mRNAs) is a pivotal aspect of biomolecules, tightly linked to gene regulation and protein synthesis, and offers innovative insights into disease diagnosis and drug development in the field of biomedicine. Several computational methods have been proposed to predict the subcellular localization of mRNAs within cells. However, there remains a deficiency in the accuracy of these predictions. In this study, we propose an mRCat predictor based on the gradient boosting tree algorithm specifically to predict whether mRNAs are localized in the nucleus or in the cytoplasm. This predictor firstly uses large language models to thoroughly explore hidden information within sequences and then integrates traditional sequence features to collectively characterize mRNA gene sequences. Finally, it employs CatBoost as the base classifier for predicting the subcellular localization of mRNAs. The experimental validation on an independent test set demonstrates that mRCat obtained accuracy of 0.761, F1 score of 0.710, MCC of 0.511, and AUROC of 0.751. The results indicate that our method has higher accuracy and robustness compared to other state-of-the-art methods. It is anticipated to offer deep insights for biomolecular research.

## 1. Introduction

Several studies have suggested that the intracellular localization of RNA plays a role in cellular function mechanisms [1,2,3]. In the regulation process of gene expression, the subcellular localization of messenger RNAs (mRNAs) is a crucial step. This process exerts precise control of the site of protein synthesis, thereby influencing the expression and regulation of relevant cellular functions [4,5,6,7]. The subcellular localization of mRNAs is primarily governed by a dynamic regulation of transport mechanisms between the cell nucleus and the cytoplasm [8]. Once mRNAs are synthesized within the cell nucleus and undergo appropriate RNA splicing, they must traverse the nuclear membrane through the nuclear pore complex to enter the cytoplasm. This nuclear translocation process is one of the key regulatory steps in gene expression, as it determines the distribution of mRNAs in the cytoplasm [9,10,11,12]. Furthermore, the subcellular localization of mRNAs plays a crucial role in pharmaceutical applications, particularly in areas such as vaccine development, gene therapy, and drug discovery [13,14,15]. mRNA vaccines represent a novel type of vaccines that utilize mRNA molecules containing encoded specific antigens to induce the immune system to produce antibodies [16]. In vaccine development, the precise control of mRNA subcellular localization is paramount to ensure the effective expression and presentation of the synthesized proteins (antigens) to the immune system. By modulating mRNA localization within the cytoplasm, vaccine researchers can optimize antigen expression and the intensity of the immune responses, thereby enhancing vaccine efficacy. mRNAs can serve as a tool for delivering therapeutic information in gene therapy [17,18,19]. By modulating mRNA localization within cells, researchers can achieve the targeted expression of therapeutic genes, thereby more precisely controlling treatment outcomes. For example, by introducing mRNAs encoding therapeutic genes into the cell nucleus or cytoplasm, gene expression and protein synthesis can be more effectively achieved in gene therapy. In the process of drug development, the subcellular localization of mRNAs plays a critical role in the study of synthesizing drugs or drug targets [20]. By adjusting the intracellular localization of mRNAs, the synthesis location of specific proteins can be influenced, thereby regulating related biological processes. Therefore, understanding the subcellular localization of mRNAs is of significant importance for studying its functionality.

Presently, the subcellular localization of RNA is primarily studied through wet-lab experiments and computational methods. RNA fluorescence in situ hybridization (FISH) enables the accurate detection of RNA subcellular localization at single-RNA resolution and in live cells [21,22,23]. However, wet-lab experiments typically require specific experimental conditions and materials and significant time investment, which makes them not only expensive but also time-consuming. In contrast, computational methods leverage computer algorithms and mathematical models to efficiently analyze large-scale datasets and recognize patterns, thereby predicting RNA subcellular localization. The advantages of this approach lie in its high throughput, automation, and relatively lower costs. Various computational methods have been applied to determine RNA subcellular localization, such as mRNALoc [24], SubLocEP [5], RNATracker [25], and RNAlight [26]. The mRNALoc model was used to investigate the localization of mRNAs within both the cytoplasm and the nucleus. It was accomplished by employing a support vector machine (SVM) [27] to analyze sequence data and predict the subcellular localization of mRNAs. The SubLocEP is a two-layer integrated prediction model leveraging comprehensive feature attributes and LightGBM [28] classifiers, demonstrating superior accuracy and generalization on independent datasets for eukaryotic mRNA subcellular localization prediction. The RNATracker is a computational method developed based on an LSTM recurrent neural network with attention mechanism and integrates mRNA sequence and secondary structure information encoded by 4 bit and 6 bit one-hot encoding or mRNA subcellular localization prediction. RNAlight is a machine learning model based on LightGBM, designed to identify nucleotide k-mers that contribute to mRNA subcellular localization, demonstrating excellent performance in determining mRNA subcellular localization. The utilization of these computational methods provides powerful tools and resources for research into mRNA subcellular localization. However, these computational methods, which primarily rely on handcrafted features represented in a traditional manner, do not fully capture the complex and ill-defined patterns and relationships within biological sequences. For instance, nucleotide k-mer features focus solely on the local characteristics of a sequence, neglecting the order of nucleotides beyond the length of the k-mer used and thus losing the global features. Therefore, this method limits the comprehensive understanding of RNA gene sequences, and the accuracy of mRNA subcellular localization prediction still needs improvement.

In this study, we propose a new machine learning-based subcellular localization predictor for mRNAs, named mRCat. It is worth noting that in this study, only two primary subcellular localizations, the nucleus and the cytoplasm, were utilized for model training and prediction. mRNAs in the cytosol, insoluble, and membrane fractions of general cytoplasmic extracts were considered to be located in the cytoplasm, and mRNAs in the nucleus, nucleolus, lamina, and nuclear pore were considered to be located in the nucleus. This predictor initially harnesses large language models to deeply explore the implicit information within a sequence. It then amalgamates traditional sequence characteristics for a comprehensive portrayal of mRNA gene sequences. Ultimately, it utilizes CatBoost [29] as the foundational classifier to predict the subcellular localization of mRNAs. We performed 100-time 5-fold cross-validation to evaluate the model performance. Additionally, to further validate the reliability of the model, we utilized an independent dataset for additional validation, assessing the model’s performance on unseen data. The results indicate that large language models can characterize mRNA gene sequences more effectively than traditional methods, and compared to other state-of-the-art approaches, our method showed higher accuracy and robustness.

## 2. Materials and Methods

### 2.1. Datasets

High-quality datasets are essential for constructing robust models. This study constructed a prediction model for mRNA subcellular localization based on the dataset established by Guo-Hua Yuan et al. [26]. CeFra-seq [30] is a technique used to study RNA–protein interactions. This technique involves crosslinking RNA with proteins in live cells, followed by separating the crosslinked complexes into different subcellular structures. Ultimately, high-throughput sequencing is employed to reveal the subcellular localization information of RNA. APEX-Seq [31] is a technique used to investigate the localization of RNA within subcellular structures. It integrates protein biology and localization analysis, accurately marking the positions of RNA within subcellular compartments using protein tags. The subcellular localization information of mRNAs is identified through datasets generated by two advanced techniques, CeFra-seq and APEX-Seq. Considering variations under different conditions (e.g., different cell lines or experimental methods), some RNAs exhibit multiple inconsistent localizations in publicly available datasets. Given this inconsistency, further data processing was conducted, including the removal of RNAs inconsistent with multiple localizations [30]. After this filtering step, we obtained a comprehensive dataset, comprising 2256 mRNA sequences located in the nucleus and 2924 mRNA sequences located in the cytoplasm. Finally, the dataset was divided into training and testing sets at a ratio of 9:1. The training set consisted of 4662 mRNA gene sequences, with 2028 mRNA sequences located in the nucleus and 2634 mRNA sequences located in the cytoplasm. The testing set comprised 518 mRNA gene sequences, with 228 mRNA sequences located in the nucleus and 290 mRNA sequences located in the cytoplasm. The testing set was used as an independent test set for independent testing, and 5-fold cross-validation was conducted on the training set.

### 2.2. Framework of mRCat

**Figure 1:**
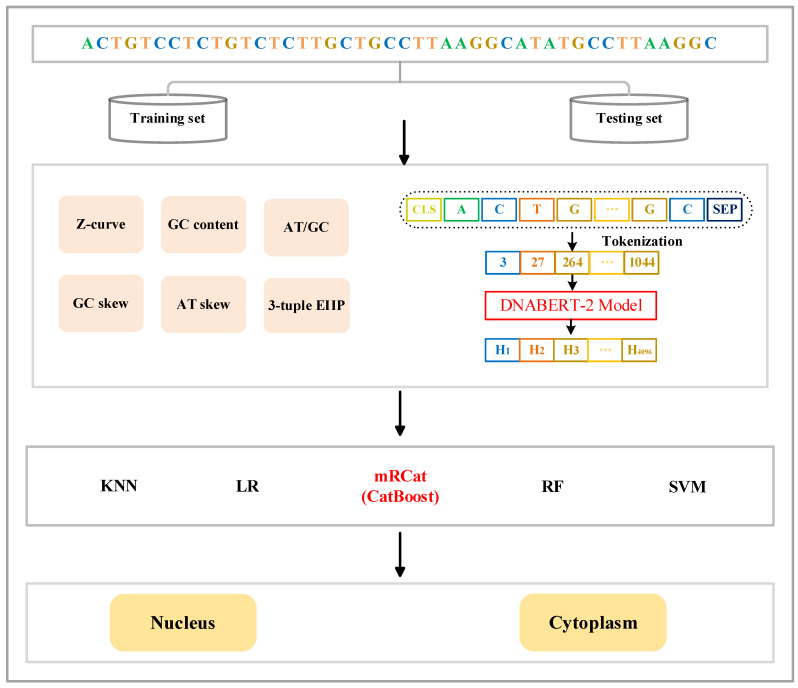
Framework of mRCat. mRCat consists of the three modules of feature encoding, classifier selection, and subcellular localization prediction. The feature encoding module combines two different feature encoding methods: traditional manual encoding and large language model encoding. Traditional manual encoding includes Z-curve, GC content, AT/GC ratio, GC skew, AT skew, and 3-tuple ElIP. Encoding in the large language model utilizes DNABERT-2. Ultimately, the sequences are represented as feature vectors. The classifier selection module involves choosing from five different classifiers: KNN, LR, CatBoost, RF, and SVM. CatBoost was ultimately chosen as the base classifier for mRCat. The subcellular localization prediction module utilizes the base classifier for subcellular localization prediction.

We constructed an mRCat model based on the CatBoost framework for mRNA subcellular localization and compared its performance with a series of other machine learning algorithms. The specific workflow is depicted in Figure 1. The machine learning algorithms we employed included K-nearest neighbor (KNN) [32], logistic regression (LR) [33], random forest (RF) [34], and support vector machine (SVM). When handling both the training and the testing sets, we utilized the same feature encoding method and represented features using both traditional handcrafted features and learned features from large language models. Finally, we integrated these two types of features into the model training and the evaluation process. We utilized library functions from the scikit-learn [35] standard machine learning library to train and test the model in Jupyter (iPython) Notebooks. The training and testing sets can be obtained from https://zenodo.org/records/12044998 (accessed on 18 June 2024)

The CatBoost algorithm, a variant of gradient boosting, is the third iteration in the GBDT family following XGBoost [36] and LightGBM. It specializes in solving classification and regression problems. CatBoost utilizes the gradient boosting tree algorithm for model training, iteratively fitting a series of weak classifiers (typically, decision trees). Each iteration trains a new classifier based on the residuals from the previous round, gradually enhancing the model performance. Additionally, we employed early stopping during training. Training halted when the model failed to show performance improvement within 50 rounds, conserving unnecessary training time and computational resources, and preventing overfitting to the training data, thereby enhancing the model generalization. Considering these advantages, along with CatBoost’s superior classification capabilities compared to other machine learning algorithms, we chose CatBoost as the base classifier for the model framework to train.

### 2.3. Feature Encoding

#### 2.3.1. Nucleotide Composition

To obtain feature information from mRNA gene sequences, we started by analyzing their nucleotide composition [37]. In this study, five calculation methods were employed: *Z-curve*, *GC content*, *AT/GC ratio*, *GC skew*, and *AT skew*. The calculation formulas are as follows:

(1) $$Z-Curve=\left\{\begin{array}{c} X\;=\;\left(F_{A}+F_{G}\right)-\left(F_{C}+F_{T}\right)\\ Y\;=\;\left(F_{A}+F_{C}\right)-\left(F_{G}+F_{T}\right)\\ Z\;=\;\left(F_{A}+F_{T}\right)-\left(F_{G}+F_{C}\right) \end{array}\right.$$

(2) $$GC\;content=\frac{F_{G}+F_{C}}{F_{A}+F_{T}+F_{G}+F_{C}}$$

(3) $$AT/GC=\frac{F_{A}+F_{T}}{F_{G}+F_{C}}$$

(4) $$GCskew=\frac{F_{G}-F_{C}}{F_{G}+F_{C}}$$

(5) $$ATskew=\frac{F_{A}-F_{T}}{F_{A}+F_{T}}$$

where $F_{X}$ represents the frequency of occurrence of the bases *ATGC* in the gene sequence.

It is noteworthy that we could directly extract the aforementioned feature information using the PyFeat (1.0) [38] tool.

#### 2.3.2. Three-Tuple Nucleotide Electron–Ion Interaction Pseudopotential

The energy of delocalized electrons in amino acids and nucleotides is calculated as Electron–Ion Interaction Pseudopotential (EIIP) [39]. In this approach, we employed *k*-mers and Electron–Ion Interaction Pseudopotentials (EIIPs) to characterize mRNA gene sequences. *k*-mers are crucial in biological sequence analysis, and the calculation of *k*-mer frequencies is considered an important method for extracting local features of gene sequences. An RNA gene sequence of length *L* contains *L* − *k* + 1 *k*-mer subsequences, with 4*^k^* possible *k*-mer subsequences. An RNA gene sequence of length *L* is assumed to be as follows:

(6) $$S=N_{1},N_{2},N_{3},N_{4},N_{5},N_{6},N_{7},N_{8},N_{9},\cdot \cdot \cdot ,N_{L-1},N_{L}$$

where *N_j_* represents any one of the nucleotides ATGC.

In this approach, we utilized 3-tuple nucleotides. When *k* is 3, the set of nucleotide compositions for a sequence is as follows:

(7) $$T=\left\{N_{1}N_{2}N_{3},N_{2}N_{3}N_{4},N_{3}N_{4}N_{5},N_{4}N_{5}N_{6},\cdot \cdot \cdot ,N_{L-2}N_{L-1}N_{L}\right\}$$

where *N_j_* represents any one of the nucleotides ATGC.

The set *T* contains a total of *L* − 3 + 1 subsequences. When *k* is 3, there are a total of 4^3^ = 64 possible *k*-mer subsequences, such as the set *W* = {*AAA*, *AAT*, *AAG*, *AAC*, …, *CCC*}. Therefore, the 3-mer features of this RNA gene sequence can be represented by a 64-dimensional vector as follows:

(8) $$V=\left[X_{1},X_{2},X_{3},X_{4},\cdot \cdot \cdot ,X_{64}\right]$$

where *Xi* represents the frequency of occurrence of each *k*-mer subsequence in set *T*, and each *k*-mer subsequence is from set *W*.

The EIIP values for each nucleotide are shown in Table 1. The sum of the EIIP values of each nucleotide in the 3-tuple was calculated as the EIIP value of the 3-tuple. Finally, the EIIP value of the 3-tuple was multiplied by its frequency to construct a 64-dimensional vector to characterize the mRNA gene sequence.

#### 2.3.3. Large Language Model Features

Relying solely on handcrafted features may lead to the loss of positional information for nucleotides in gene sequences. To address this, we introduced the pre-trained language model DNABERT-2 [40]. DNABERT-2 is a multi-species genomic base model tokenized using Byte Pair Encoding (BPE) [41], employing multiple strategies to overcome input length restrictions, reduce time and memory consumption, and enhance model capacity. Specifically, DNABERT-2 employs Attention with Linear Biases (ALiBi) [42] as a replacement for the conventional positional embeddings [43] utilized by mainstream language models to overcome input length limitations, coupled with Flash Attention [44] to increase the computational efficiency.

DNABERT-2 represents an iterative advancement of DNABERT [43], adapting the transformer encoder architecture similar to BERT [45], and contains 12 transformer layers. Each layer is equipped with 768 hidden units and 12 attention heads. DNABERT-2 first employs BPE to tokenize mRNA gene sequences as input, and each sequence is represented as a matrix M by embedding each token into a numerical vector. Subsequently, it utilizes a multi-head self-attention mechanism to capture contextual information on M:

(9) $$MultiHead\left(M\right)=Concat\left({head}_{1},{head}_{2},{head}_{3},\ldots ,{head}_{h}\right)W^{O}$$

(10) $${head}_{i}=softmax\left(\frac{MW_{i}^{Q}MW_{i}^{K}T}{\sqrt{d_{k}}}\right)\cdot MW_{i}^{V}$$

where $W^{O}$, $W_{i}^{Q}$, $W_{i}^{K}$, and $W_{i}^{V}{\left\{W_{i}^{Q},W_{i}^{K},W_{i}^{V}\right\}}_{i=0}^{h}$ are learned parameters for linear projection.

Head calculates the next hidden states of M by first computing the attentions scores between every two tokens and then utilizing them as weights to sum up lines in $MW_{i}^{V}\cdot MultiHead()$ concatenates the results of each independent head with different sets of $\left\{W_{i}^{Q},W_{i}^{K},W_{i}^{V}\right\}$. The iteration occurs L times, aligning with the number of layers within the model architecture.

To address the challenge of explicit positional information required in attention-based models, DNABERT-2 integrates Attention with Linear Biases (ALiBi) within its multi-head self-attention mechanism. Rather than adding positional embeddings to the input, ALiBi incorporates positional information into attention scores by introducing a fixed set of static, non-learned biases to each attention calculation.

The sequence tokens undergo processing through 12 transformer blocks, each of which incorporates multi-head self-attention mechanisms. Subsequently, we extracted the mean value of the first output from the last hidden state to represent the mRNA gene sequence information for mRNA subcellular localization. Each sequence is represented as a 768-dimensional feature vector.

### 2.4. Performance Evaluation Metrics

We adopted a systematic and comprehensive model evaluation approach to ensure the accurate and reliable assessment of the proposed model’s performance in addressing the specific problem. The model was primarily evaluated using the following five metrics:

(11) $$Accuracy=\frac{TP+TN}{TP+TN+FP+FN}$$

(12) $$Recall=\frac{TP}{TP+FN}$$

(13) $$Precision=\frac{TP}{TP+FP}$$

(14) $$F1score=\frac{2\times Precision\times Recall}{Precision+Recall}$$

(15) $$MCC=\frac{TP\times TN-FP\times FN}{\sqrt{\left(TP+FP\right)\left(TP+FN\right)\left(TN+FP\right)\left(TN+FN\right)}}$$

where TP refers to instances where the model correctly classifies positive samples as positive, TN refers to instances where the model correctly classifies negative samples as negative, FP refers to instances where the model incorrectly classifies negative samples as positive, and FN refers to instances where the model incorrectly classifies positive samples as negative.

## 3. Results and Discussion

### 3.1. Comparison of Different Classifiers

**Figure 2:**
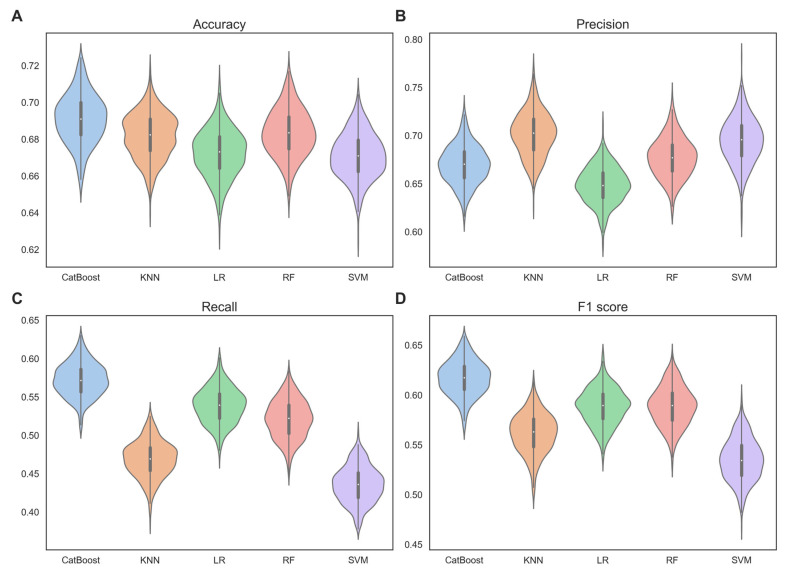
Violin plots, showing the performance of each base classifiers in terms of (**A**) accuracy, (**B**) precision, (**C**) recall, and (**D**) F1 score, were verified by 100-time 5-fold cross-validation on the training set.

Cross-validation is a commonly used technique for model evaluation, based on the principle of slicing a dataset into different training and validation sets [46]. In this study, we performed 100-time 5-fold cross-validation on the training set, comparing the mean and standard deviation of the performance metrics for different base classifiers. Figure 2 shows the performance of each base classifiers in terms of accuracy, precision, recall, and F1 score.

From Figure 2, we can observe that CatBoost significantly outperformed the other base classifiers. Specifically, the mean accuracy of CatBoost was 0.691, which was higher than the mean accuracy of KNN (0.682), LR (0.672), RF (0.683), and SVM (0.671) by 1.3%, 2.8%, 1.2% and 2.9%, respectively. The mean F1 score of CatBoost was 0.617, which was higher than the mean F1 score of KNN (0.562), LR (0.588), RF (0.589), and SVM (0.535) by 9.8%, 4.9%, 4.8%, and 15.3%, respectively. The mean precision and recall also followed the same trend. This indicates that CatBoost has superior classification performance. This may be due to CatBoost’s use of a symmetric tree structure, which ensures that each feature has the opportunity to be used at every split point in the tree, thereby better capturing nonlinear relationships in the data. The standard deviations of accuracy, precision, and recall with CatBoost (0.013, 0.020, 0.022) were comparable to those with KNN (0.012, 0.023, 0.023), LR (0.013, 0.019, 0.023), RF (0.013, 0.021, 0.025), and SVM (0.012, 0.024, 0.023). However, the standard deviation of the F1 scores with CatBoost (0.017) was lower than those obtained with KNN (0.020), LR (0.018), RF (0.020), and SVM (0.021). This suggests that CatBoost exhibits robustness and stability.

Through observation of the 100-time 5-fold cross-validation results, we discovered that CatBoost demonstrates very strong capability and robustness in mRNA subcellular localization. Therefore, choosing CatBoost as the base classifier for our mRCat model architecture was an excellent decision.

### 3.2. Comparison of Different Encoding Schemes

Utilizing effective feature representations in sequence-based prediction tasks was demonstrated to enhance model performance. In particular, in gene sequence analysis, effective features can identify specific sequence patterns and motifs. To achieve a more effective representation of mRNA gene sequence features, we employed both handcrafted features (HFs) and learned features (LFs) by large language models for sequence characterization. Traditional handcrafted feature encodings, including Z-curve, GC content, AT/GC ratio, GC skew, AT skew, and 3-tuple ElIP, are predominantly based on the nucleotide composition and the physical properties of nucleotides within mRNA gene sequences. Learned features, which are obtained through the deep learning of mRNA gene sequences by the DNABERT-2 large language model that has been pre-trained on an extensive corpus of genetic sequences, capture the interrelationships between motifs and the underlying patterns among the nucleotides that constitute a sequence. The performance metrics of CatBoost combined with various types of features are shown in Table 2. We observed that learned features (LFs) by large language models exhibited a stronger representational capacity compared to traditional handcrafted features (HFs). CatBoost + LFs obtained accuracy of 0.724 and F1 score of 0.659, while CatBoost + HFs obtained accuracy of 0.714 and F1 score of 0.649. This is because large language models are capable of learning long-distance dependencies and complex biological patterns, thereby enhancing the accuracy of predictions. Furthermore, our observations indicated that the fusion of handcrafted features with learned features facilitated a more robust characterization of mRNA gene sequences. mRCat obtained accuracy of 0.761 and F1 score of 0.710. These results demonstrate that large language models excel at learning sequence information and underscore the superiority of our model architecture.

**Figure 3:**
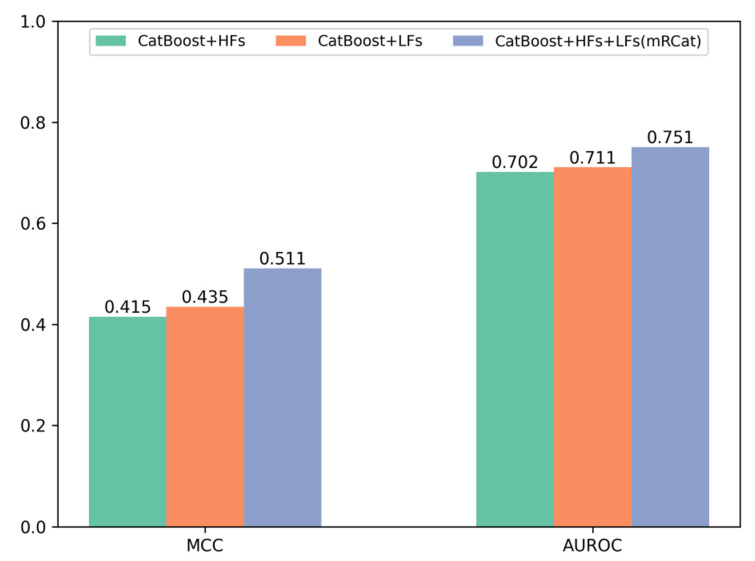
Comparison of Matthews’ correlation coefficient (MCC) and area under the receiver operating characteristic curve (AUROC) performance of different features on the independent test set.

To further demonstrate the advantages of mRCat and large language models in capturing sequence information, we conducted a comparative analysis on the performance metrics of Matthew’s correlation coefficient (MCC) and area under the receiver operating characteristic curve (AUROC) for traditional handcrafted features (HFs), features learned (LFs) by large language models, and the combination of HFs and LFs. The experimental results in Figure 3 show that large language models provide superior representation of mRNA gene sequences compared to traditional methods. Additionally, combining HFs with LFs enabled a more comprehensive learning of sequence characteristics. Specifically, on an independent test set, the MCC and AUROC of LFs surpassed those of HFs individually. Moreover, the combination of HFs and LFs in mRCat demonstrated significantly higher MCC and AUROC compared to using HFs or LFs alone. On average, mRCat showed a 23.1% increase in MCC compared to HFs and a 17.5% improvement compared to LFs. Additionally, mRCat demonstrated a 7% increase in AUROC over HFs and a 5.63% increase over LFs. The integration of handcrafted and learned features capitalizes on the advantages of each. Handcrafted features grant a clear and interpretable insight into the data, enhancing our understanding of the underlying mechanisms. In contrast, learned features, particularly those derived from large language models, excel at discerning subtle and complex patterns that may not be immediately apparent. This synergistic approach allows for a more comprehensive and nuanced analysis, ultimately improving the predictive power and interpretability of models. The comprehensive results substantiate the superior capability of mRCat in learning sequence information.

### 3.3. Comparison with Other Predictors

To further evaluate the performance of mRCat in predicting mRNA subcellular localization, we compared mRCat with several existing state-of-the-art predictors by using an independent test set. From Table 3, we can observe that mRCat significantly outperformed existing predictors. Specifically, mRCat consistently surpassed other existing predictors across all performance metrics. mRCat obtained accuracy of 0.761 and F1 score of 0.710.

## 4. Conclusions

The subcellular spatial distribution pattern of mRNAs represents a pivotal avenue for elucidating mRNA regulation, functionality, and associated pathologies, thereby furnishing crucial insights into the intricate landscape of intracellular gene expression regulatory networks. In this study, we propose mRCat, a gradient-boosted tree-based architecture for mRNA subcellular localization. To identify an exceptional base classifier for mRCat, we conducted 100-time 5-fold cross-validation on five classifiers: k-nearest neighbor (KNN), logistic regression (LR), random forest (RF), support vector machine (SVM), and CatBoost. This rigorous evaluation was performed to gauge the classifiers’ performance on the training sets, thus informing the selection of the most suitable base classifier for mRCat. Through observation of the 100-time 5-fold cross-validation results, we discovered that CatBoost demonstrated the strongest capability in mRNA subcellular localization. Therefore, choosing CatBoost as the base classifier for our mRCat model architecture was an excellent decision. By effectively characterizing mRNA gene sequences, the performance of model architectures in predicting mRNA subcellular localization can be significantly enhanced. In addition to employing traditional methods such as Z-curve, GC content, AT/GC, GC skew, AT skew, and 3-tuple EIIP for sequence feature extraction, we also incorporated the advanced large language model DNABERT-2 to automatically learn latent information within the sequences. Using the independent test set, the results showed accuracy, precision, recall, and F1 score values as shown in Table 2 and MCC and AUROC as shown in Figure 3. We found that utilizing sequence features learned by large language models offered superior characterization of mRNA gene sequences compared to the use of features extracted through traditional methods. Ultimately, the fusion of handcrafted features extracted through traditional methods with learned features from the large language model resulted in a further enhancement of our model’s performance, underscoring the superiority of our feature extraction methodology.

In conclusion, the subcellular spatial distribution pattern of mRNAs serves as a critical foundation for understanding mRNA regulation, functionality, and associated pathologies. Our proposed model, mRCat, leverages a gradient boosted tree-based architecture to accurately predict mRNA subcellular localization. Through 100-time 5-fold cross-validation, CatBoost was selected as the optimal classifier for our model architecture. Additionally, the integration of advanced techniques like DNABERT-2 further enhanced the model’s predictive capabilities by automatically capturing latent information within mRNA sequences. Overall, our findings underscore the importance of leveraging both traditional and state-of-the-art methodologies to advance our understanding of mRNA biology and subcellular localization dynamics.

## Figures and Tables

**Table 1:** Electron–ion interaction pseudopotentials of nucleotides.

Nucleotide	EIIP
A	0.1260
C	0.1340
G	0.0806
T	0.1335

**Table 2:** Effects of different features on the predictive performance of mRNA subcellular localization on the independent test set.

	Evaluation Metrics			
Model	Accuracy	Precision	Recall	F1 Score
CatBoost + HFs	0.714	0.706	0.601	0.649
CatBoost + LFs	0.724	0.723	0.605	0.659
CatBoost + HFs + LFs (mRCat)	0.761	0.760	0.667	0.710

**Table 3:** Comparison with state-of-the-art models on the independent test set.

	Evaluation Metrics			
Model	Accuracy	Precision	Recall	F1 Score
mRCat (CatBoost)	0.761	0.760	0.667	0.710
RNAlight	0.73	0.75	0.59	0.66
SubLocEP	0.655	0.66	0.65	0.655
RNATracker	0.516	0.595	0.519	0.554
mRNALoc	0.591	0.545	0.49	0.516

## Data Availability

The source codes and data for mRCat are available at https://zenodo.org/records/12044998 (accessed on 18 June 2024).
